# Latest developments in the treatment of lung cancer

**DOI:** 10.1186/1753-6561-2-s2-s1

**Published:** 2008-09-24

**Authors:** Aldo Castagnari

**Affiliations:** 1 Centro Integral de Oncología. Leandro N. Alem 460 (1832) Lomas de Zamora, Provincia de Buenos Aires, Argentina

## 

An Experts Meeting on Lung Cancer and the Use of Erlotinib was held on 10^th ^November 2007, in New York City, USA. The meeting was organized by ACINDES (*Asociación Civil de Investigación y Desarrollo en Salud *[Civil Association for Health Research and Development]), a private international academic institution actively involved in medical research and the development of educational programs since 1985.

The meeting brought together a group of distinguished lung cancer oncologists with the aim of reviewing existing evidence on the use of erlotinib for the treatment of this pathology. Dr. Néstor Mendyk was in charge of opening the event, which was coordinated by the oncology specialist Dr. Aldo Castagnari. Also attending the meeting were Dr. Víctor Lira Puerto, medical oncologist at the ABC Hospital, Mexico City, and, as a special guest, Dr. Nicholas Thatcher, Professor of Medical Oncology at Manchester University, UK, who offered lectures with up-dated information on the use of erlotinib.

The agenda and the most relevant information were developed using a series of questions as a guide so that participating experts could exchange data from their own experience and published literature. These questions included addressed the most relevant information on efficacy and safety regarding erlotinib use. This publication is a most important tool for the Latin America medical community because it will provide access to a summary of the lectures and to the most prominent opinions of regional professionals and experts in the field.

## Competing interests

Dr. Aldo Castagnari declares that he has no competing interests.

